# Supplementary material for: Diachronic and synchronic variation in the performance of adaptive machine learning systems: the ethical challenges
Source: J Am Med Inform Assoc. 2022 Nov 15;30(2):361–6. doi: 10.1093/jamia/ocac218 (PMC9846684; doi:10.1093/jamia/ocac218)
Supplement: ocac218_Supplementary_Data [file ocac218_supplementary_data.zip › ocac218_Supplementary_Data/CoverPage.docx]

### Article and Author Information

*Title*: Diachronic and synchronic variation in the performance of adaptive machine learning systems: The ethical challenges

*Corresponding author:* Joshua Hatherley

Philosophy Department

School of Philosophical, Historical and International Studies
Monash University
Level 6, 20 Chancellor's Walk (Menzies Building)
Wellington Road
Clayton VIC 3800
Australia

+613 9905 8735
[joshua.hatherley@monash.edu](mailto:joshua.hatherley@monash.edu)

*Other author:*  Robert Sparrow

Philosophy Department

School of Philosophical, Historical and International Studies
Monash University
Level 6, 20 Chancellor's Walk (Menzies Building)
Wellington Road
Clayton VIC 3800
Australia

*Keywords:* artificial intelligence, bioethics, update problem, medicine, federated learning

*Manuscript length:* 3,968 words
