# Supplementary material for: Diachronic and synchronic variation in the performance of adaptive machine learning systems: the ethical challenges
Source: J Am Med Inform Assoc. 2022 Nov 15;30(2):361–6. doi: 10.1093/jamia/ocac218 (PMC9846684; doi:10.1093/jamia/ocac218)
Supplement: ocac218_Supplementary_Data [file ocac218_supplementary_data.zip › ocac218_Supplementary_Data/JAMIA_Figure.docx]

**Space (x)**

**T^0^**

**T^1^**

**T^2^**

**Site *1***

**Site *2***

MAMLS^1^

MAMLS^3^

MAMLS^5^

MAMLS^1^

MAMLS^2^

MAMLS^4^

**Time (y)**
